# Leveraging orthology within maize and Arabidopsis QTL to identify genes affecting natural variation in gravitropism

**DOI:** 10.1073/pnas.2212199119

**Published:** 2022-09-26

**Authors:** Takeshi Yoshihara, Nathan D. Miller, Fernando A. Rabanal, Hannah Myles, Il-Youp Kwak, Karl W. Broman, Boris Sadkhin, Ivan Baxter, Brian P. Dilkes, Matthew E. Hudson, Edgar P. Spalding

**Affiliations:** ^a^Department of Botany, University of Wisconsin, Madison, WI 53706;; ^b^Department of Molecular Biology, Max Planck Institute for Biology Tübingen, 72076 Tübingen, Germany;; ^c^Department of Biostatistics and Medical Informatics, University of Wisconsin, Madison, WI 53706;; ^d^Carl R. Woese Institute for Genomic Biology, University of Illinois at Urbana–Champaign, Urbana, IL 61801;; ^e^Donald Danforth Plant Science Center, St. Louis, MO 63132;; ^f^Department of Biochemistry, Purdue University, West Lafayette, IN 47907;; ^g^Department of Crop Sciences, University of Illinois at Urbana–Champaign, Urbana, IL 61801;; ^h^Department of Applied Statistics, Chung-Ang University, Seoul 06974, Republic of Korea

**Keywords:** gravitropism, high-throughput phenotyping, QTL, orthology

## Abstract

Plants typically orient their organs with respect to the Earth’s gravity field by a dynamic process called gravitropism. To discover conserved genetic elements affecting seedling root gravitropism, we measured the process in a set of *Zea mays* (maize) recombinant inbred lines with machine vision and compared the results with those obtained in a similar study of *Arabidopsis thaliana*. Each of the several quantitative trait loci that we mapped in both species spanned many hundreds of genes, too many to test individually for causality. We reasoned that orthologous genes may be responsible for natural variation in monocot and dicot root gravitropism. If so, pairs of orthologous genes affecting gravitropism may be present within the maize and *Arabidopsis* QTL intervals. A reciprocal comparison of sequences within the QTL intervals identified seven pairs of such one-to-one orthologs. Analysis of knockout mutants demonstrated a role in gravitropism for four of the seven: *CCT2* functions in phosphatidylcholine biosynthesis, *ATG5* functions in membrane remodeling during autophagy, *UGP2* produces the substrate for cellulose and callose polymer extension, and *FAMA* is a transcription factor. Automated phenotyping enabled this discovery of four naturally varying components of a conserved process (gravitropism) by making it feasible to conduct the same large-scale experiment in two species.

## Significance

Plants use the Earth’s gravitational field to orient their growth, but there are large gaps in our understanding of the mechanism. We measured thousands of corn seedling roots, changing their growth direction in response to gravity (gravitropism) with machine vision methods, and we used genetic information about the seedlings to coarsely map the genomic positions of influential genes. Because we had previously performed the same experiment with the distantly related *Arabidopsis* plant, we could use a gene-relatedness search to pinpoint only those genes residing within the relevant regions of the genome in both species. Follow-up tests verified the identity of four genes that modify root gravitropism. The new information could help us understand how gravity shapes root system architectures.

Gravity provides a constant guide for plant growth on Earth. A plant determines the direction of the gravity vector primarily by registering where dense plastids called statoliths settle in some endodermal cells of shoots and cap cells of roots (1). In root cap cells, statolith sedimentation triggers an intracellular redistribution of PIN auxin transporters (2), which diverts intercellular auxin transport more to the lower side (3), where it slows the rate of cell expansion possibly by increasing apoplastic pH (4, 5). The resulting growth differential produces curvature that reorients the root tip.

Various experimental approaches have identified molecules that play a role in gravitropism (6). In addition to the core components of a gravity-signaling mechanism, which presently cannot be described without major gaps, there are probably ancillary regulatory modules that affect the sensitivity of the system. One experimental approach to identifying the missing components and regulators of gravitropism is to measure gravity responses in many members of a natural population of plants. If variation in the genome of each member of the population is sufficiently characterized with markers, statistical analysis methods may identify regions that are associated with variation in the phenotype. Such quantitative trait locus (QTL) mapping approaches have already produced useful information about root gravitropism. For example, a line of research that began by mapping a QTL controlling the root growth angle in rice led to the identification of a gene known as *DRO1*, which is related to the *LAZY* genes in *Arabidopsis* that are required for gravitropism (7–9). At least one LAZY protein becomes polarly localized with the PIN3 auxin transporter during the sensing phase (10).

Genetic approaches to studying root gravitropism benefit from machine vision methodologies that automatically make precise measurements of the process from image sequences (11). When such methods are used to measure gravitropism in genetically characterized populations, variation in tip angle time courses can be mapped to explanatory genomic loci (12, 13). Moore et al. (14) took this approach to map QTLs controlling *Arabidopsis* root gravitropism in a population of recombinant inbred lines (RILs). The genomic intervals that Moore et al. (14) identified were broad, containing either dozens or hundreds of genes. Experimental approaches to attaining greater resolution on the genetic axis include measuring the same phenotype in a fine-mapping population, measuring it in different populations of the same species to produce a so-called meta-QTL analysis (15), or measuring the effects of mutations in genes within the QTL intervals if some filtering criteria effectively highlighted potentially causal candidates. Here, we designed an approach based on the assumption that the gravitropism mechanism is conserved in all seed plants, for which there is evidence (16). If true, then gravitropism QTL in a monocot and a dicot could share genetic elements that evolution retained as naturally varying contributors to the mechanism. We chose the crop plant *Zea mays* (maize) as the monocot to compare with the previous Moore et al. (14) study of *Arabidopsis*, a dicot. Gravitropism was measured as time courses with similar high resolution in maize as it was in *Arabidopsis*. We implemented a method for finding one-to-one orthologs within the mapped *Arabidopsis* and maize QTL regions. We validated roles in gravitropism for four of the genes. We interpret the results in terms of gene functions that evolution has selected to fine-tune or modulate a fundamental aspect of land plant life.

## Results

We created a custom image-acquisition platform that made it feasible to measure maize root gravitropism in the Intermated B73 x Mo17 (IBM) population of RILs. Each computer-controlled camera in the platform acquired images every 3 min beginning when the seedlings were rotated 90° to initiate a gravitropic response (Video S1).

### Maize Root Gravitropism.

**Fig. 1.**
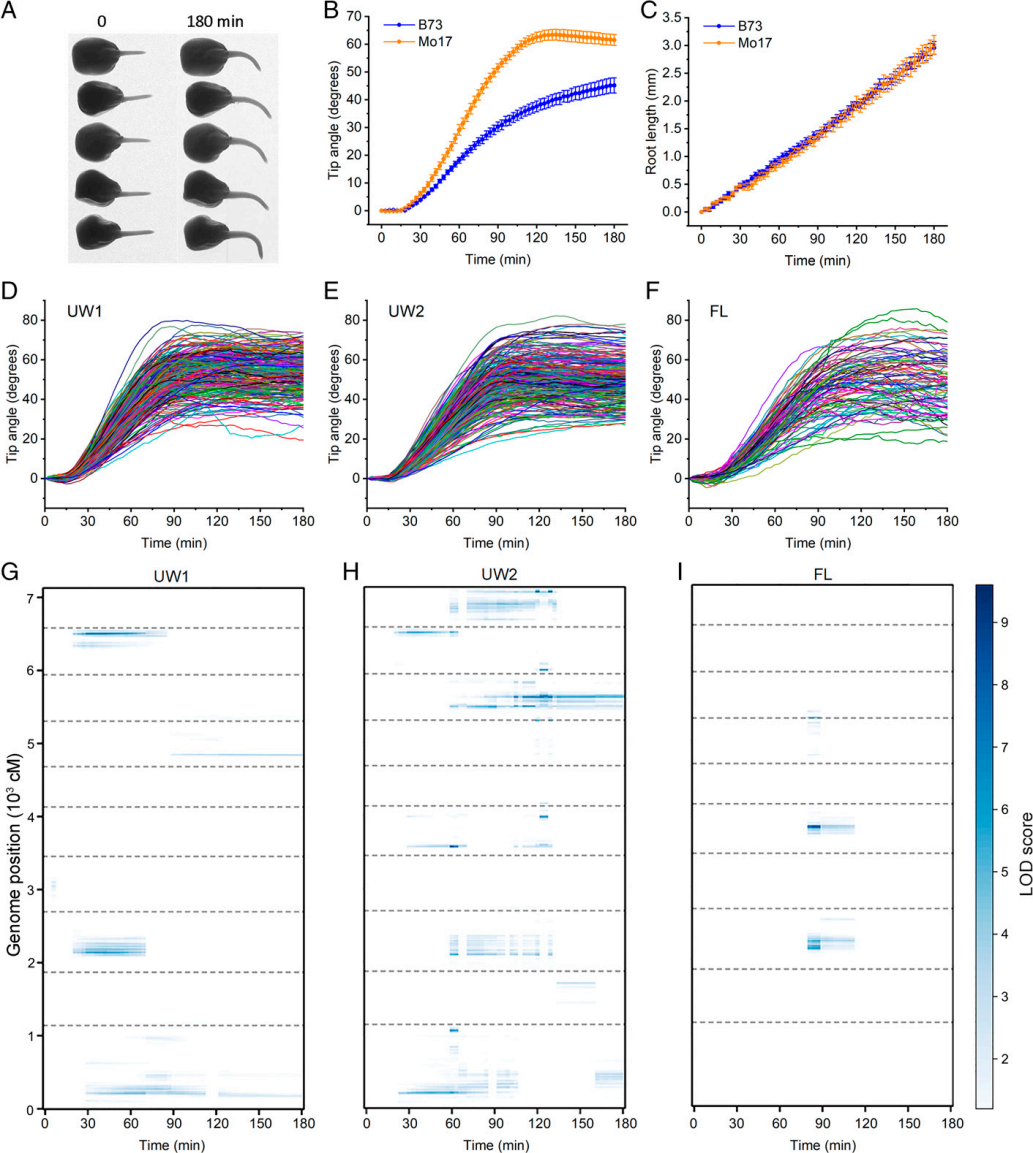
Maize root gravitropism and QTL maps. (*A*) Raw image showing five maize seedlings on one agar plate at the beginning and end of a 180-min gravitropism assay. The two images are representative of the many thousands of images automatically collected by seven cameras in parallel to measure the gravitropism responses of hundreds of maize recombinant inbred lines (RILs). (*B*) Gravitropism of the B73 and Mo17 parental lines. Tip angles were measured automatically from raw images (like those in *A*) with custom image-analysis software. In this display, the angle at t = 0 was subtracted from the angle at t > 0 to remove any initial offset from each response. Displayed are the averages of *n* = 28 roots for B73 and 17 roots for Mo17 ± SEM. (*C*) Root length minus initial root length during the gravitropism responses summarized in (*B*). The two parental lines displayed different gravitropism responses but similar elongation rates. (*D*) Average gravitropism responses of 239 IBM RILs with initial angles subtracted form a dataset called UW1. (*E*) UW2 is a dataset comprising 280 IBM RILs. (*F*) FL is a dataset comprising 89 IBM RILs sourced from the University of Florida. The average number of independent trials per RIL was 10.1 for UW1, 16.4 for UW2, and 4.3 for FL. The gravitropism curves shown in panels (*D*)–(*F*) were used to map QTL. (*G*–*I*) Time-dependent quantitative trait loci (QTL) for gravitropism were mapped with the stepwise method using tip angle (initial value subtracted) as the trait. Each time point was treated independently to produce 180 time point QTL maps, which are shown here, with LOD score represented on a blue color intensity scale, time along the x axis, and genome position along the *y* axis. The *y* axis displays cumulative centimorgans of genetic distance, with dashed lines indicating breaks between the 10 chromosomes. All coordinates with statistically insignificant LOD scores are white. (*G*) UW1, (*H*) UW2, (*I*) FL.

Fig. 1*A* shows an example image of five maize seedlings at 0 and 180 min after rotation. We developed a machine learning model to automatically identify each root tip and measure its angle for each seedling in each frame of a time series (*SI Appendix*, Method 1). The B73 and Mo17 inbred parents displayed significantly different gravitropism time courses (Fig. 1*B*) but not different elongation rates (Fig. 1*C*). Each line in Fig. 1*D* shows the average response of a different RIL. The entire population of 239 RILs produced a dataset called UW1. The experiment was repeated to produce a dataset called UW2 (Fig. 1*E*). Gravitropism was also measured in a set of fewer IBM RILs with fewer kernels per RIL obtained from the University of Florida to produce the FL dataset (Fig. 1*F*). Fig. 1 *D*–*F* shows the raw phenotype data used to map maize gravitropism QTL as a function of time.

### Mapping Gravitropism QTL over Time.

The stepwise QTL method (17) as implemented by Moore et al. (14) was used with the root tip angle at each of the 61 time points in each RIL’s mean gravitropism response curve as the trait. This produced a time series of 61 logarithm-of-odds (LOD) values for each genome position for the UW1, UW2, and FL datasets. The color-coded LOD scores as a function of genomic position and time, for each of the three datasets, show when, during gravitropism, the genotype at a particular locus marker was significantly associated with variation in root tip angle (Fig. 1 *G*–*I*). For example, Fig. 1*G* shows that in UW1 a QTL on chromosome 9 appeared ∼15 min after the initiation of gravitropism and declined within 90 min. The same QTL appeared over a similar time course in the UW2 dataset (Fig. 1*H*), but this QTL was not detected in the FL dataset (Fig. 1*I*). On chromosome 3, an early-acting, transient QTL was detected in all three datasets. The results are like those found in the similarly designed Moore et al. study (14) of *Arabidopsis* root gravitropism. *SI Appendix*, Table S1 reports the LOD scores and positions of all of the time-dependent QTL (tQTL) detected in the three maize datasets.

### Mapping Parameters of Fitted Curves.

We fit logistics and Gompertz sigmoidal functions to the mean response curve of each RIL to produce three parameters that could be mapped as quantitative traits. Lambda (λ) is a measure of the response lag phase, µ is the maximum slope, and A is the final (steady state) angle. A spline-fitting method quantified P, the peak angle in time courses that displayed an overshoot phase. Subtracting A from P produced a measure of the overshoot that we treated as a phenotype to map. We also measured the integral (I) of each fitted curve. Using these fit-based metrics as phenotypes, we mapped 14 different parameter QTLs, or pQTLs (*SI Appendix*, Table S2). The 10 tQTLs that matched pQTL were long-lasting, averaging 44 ± 9 min. The tQTL that were not also detected by parameter analysis persisted on average only 4 ± 5 min. Thus, genetic loci that affected gravitropism for many minutes were more likely to be detected by a parameter-trait analysis. Some pQTLs for µ such as Chr1-1, Chr8-1, and Chr10-1 were evident as tQTL during the curvature development phase. Variation in these loci may represent variation in the mechanism that establishes differential growth across the root. In contrast, pQTL for λ such as Chr3-1 and Chr9-2 matched tQTL that appeared early in the time course. These may reflect genetic variation in the gravity-signaling mechanism that precedes differential growth. QTL for the overshoot (P-A) trait, such as the late-appearing Chr2-1, may play a role in swinging the root tip back after peaking.

### Comparing Maize and *Arabidopsis* QTL Identifies Candidates for Orthologous Causal Genes.

The QTL intervals were broad. Two contained >1,000 gene models and 12 contained >100 (*SI Appendix*, Table S1). The analogous Moore et al. study (14) of *Arabidopsis* gravitropism also identified QTLs, each spanning many gene models. We reasoned that orthologous genes encoding conserved functions in root gravitropism may reside within our sets of maize and *Arabidopsis* QTLs. Therefore, we performed a bidirectional best hit (BBH) sequence similarity search (18) to identify one-to-one orthologs (19) within the *Arabidopsis* (14) and maize (*SI Appendix*, Table S1) gravitropism QTL intervals. From 233 *Arabidopsis* candidate genes and 5,483 maize candidate genes, the search identified only 7 BBH pairs (Table 1), a surprisingly low number. We collected mutant lines for five of these seven candidate genes and measured their gravitropism. Strikingly, four of the five genes we could study as mutants showed statistically significant gravitropism phenotypes. See *SI Appendix*, Table S3 for a summary of the sequence variations between the parental alleles (*Arabidopsis* and maize) for each of the four genes. *SI Appendix*, Table S3 also shows expression information for each of the four genes.

**Table 1.** BBH genes residing within *Arabidopsis* and maize gravitropism QTL

Maize QTL	Maize gene	*Arabidopsis* QTL	*Arabidopsis* gene	Identity (%)	Gene name	Gravitropism phenotype
Chr1-2	Zm00001eb025170	4@40.3	At4g15130	61	*CCT2*	Yes
Chr5-1	Zm00001eb216660	1@64	At1g21980	64	*PIP5K1*	No
Chr5-2	Zm00001eb230080	5@61	At5g17290	61	*ATG5*	Yes
Chr5-2	Zm00001eb230070	5@61	At5g17310	84	*UGP2*	Yes
Chr8-2	Zm00001eb344960	3@17	At3g24140	48	*FAMA*	Yes
Chr10-1	Zm00001eb423930	1@64	At1g22275	41	*ZYP1*	Not tested
Chr10-2	Zm00001eb431340	5@61	At5g16860	55	None	Not tested

Genes found within the intervals were used as reciprocal queries. *Arabidopsis* intervals are from Moore et al. (14) and maize intervals are from the present study (*SI Appendix*, Table 1). BBH, bidirectional best hit.

We conclude that natural variation in *CCT2*, *FAMA*, *UGP2*, and *ATG5* is likely to affect gravitropism in maize and *Arabidopsis* primary roots.

### *FAMA* Negatively Affects Gravitropism.

**Fig. 2.**
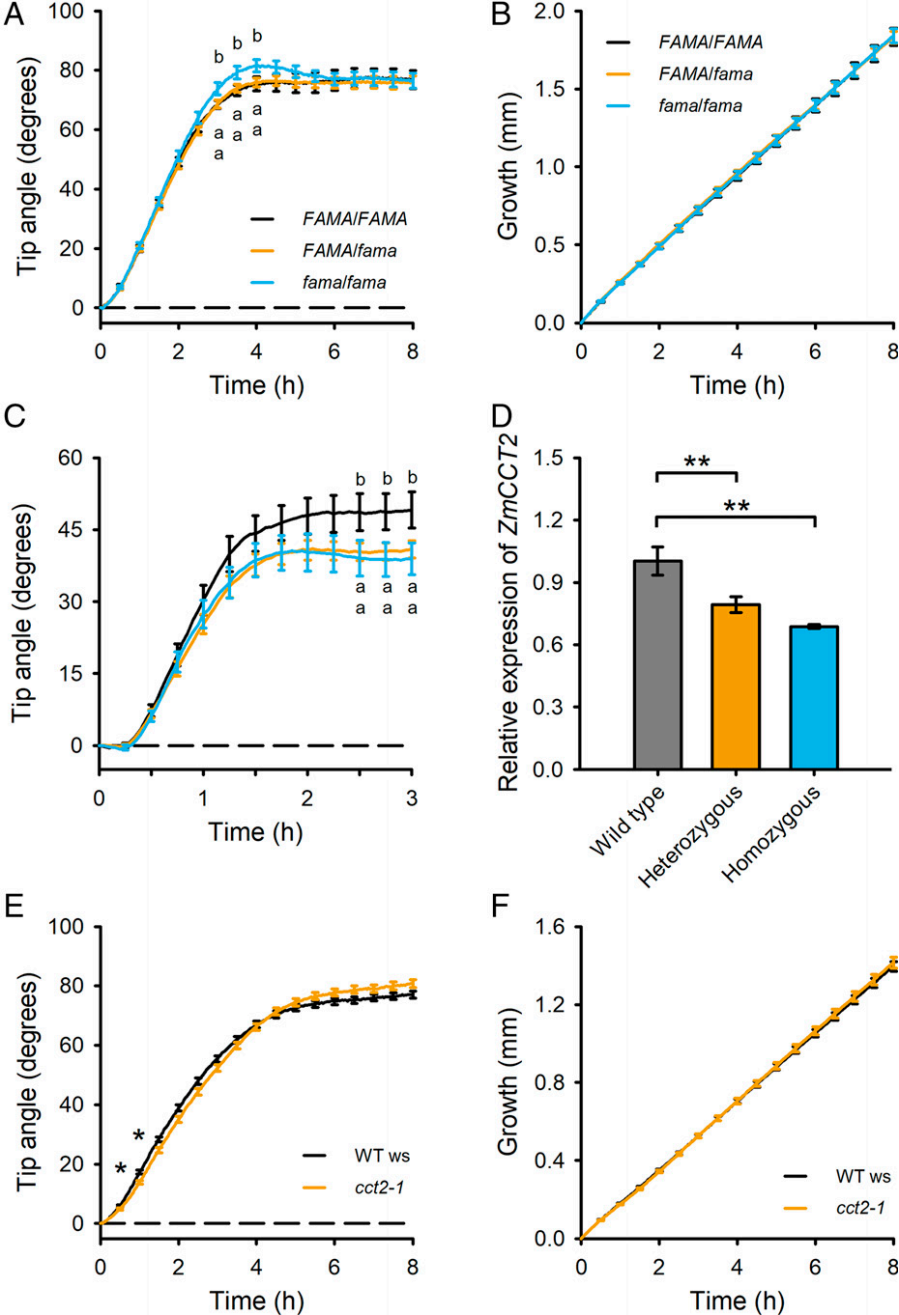
Gravitropism phenotype supports causal QTL gene status for *FAMA* and *CCT2*. (*A*) Tip angles of *Arabidopsis* primary roots following 90° rotation were measured every 2 min for 8 h. Plants segregating for the *fama* mutation were genotyped after the assay. *n* = 35 *FAMA*/*FAMA*, 66 for *FAMA*/*fama*, and 37 for *fama*/*fama* seedlings. The SEM error bars are shown at 30-min intervals instead of at each of the 240 time points for clarity. Statistically significant differences determined by one-way ANOVA with Tukey honestly significant difference (HSD) test (α = 0.1) are indicated by different letters. (*B*) Elongation of the same roots during the gravitropism response was not affected by *FAMA* genotype. (*C*) Impaired gravitropism of *cct2* mutant in maize. *n* = 19 for wild type, 54 for heterozygous, and 23 for homozygous *cct2* mutants. The genotype (Mu insertion in the promoter) was determined after the assay. (*D*) Reduced expression of *ZmCCT2* in the root tips of homozygous and heterozygous maize *cct2* seedlings. The SEM error bars are shown at 15-min intervals instead of at each of the 60 time points for clarity. (*E*) *Arabidopsis cct2* mutant gravitropism deviated slightly from the wild type. Asterisks indicate statistically significant difference from the wild type by Student’s *t* test (**P < 0.01). (*F*) Elongation did not differ from wild type to a statistically significant extent. *n* = 90 for WT and 84 for *cct2-1*.

*FAMA*, a gene within the confidence intervals of QTL Chr8-2 in maize and 3@17 in *Arabidopsis* (14), encodes a basic helix-loop-helix (bHLH) transcription factor that controls differentiation of stomata. Because homozygous *fama* mutants are sterile (20), we measured gravitropism in a segregating population. The genotype of each seedling was determined after the gravitropism response had been measured. Wild-type (*FAMA*/*FAMA*) and heterozygous (*FAMA*/*fama*) root responses were indistinguishable, but homozygous mutant siblings (*fama*/*fama*) showed an overshoot in the tip angle curve (Fig. 2*A*). The overshoot phenotype is consistent with this QTL being associated with the amplitude trait (parameter A, peak angle), and the integral trait (parameter I, area under the curve). The growth rate of *fama*/*fama* roots was the same as wild type during the gravitropism response (Fig. 2*B*). These data indicate that FAMA influences differential growth across the root to reduce the overshoot during gravitropism, a new role for this well-studied transcription factor.

### *CCT2* Positively Affects Gravitropism.

*CCT2*, a gene within the maize gravitropism QTL Chr1-2 (Table 1) and the *Arabidopsis* gravitropism QTL 4@40.3 (14) encodes a CTP:phosphorylcholine cytidylyltransferase. The reaction that this enzyme catalyzes is typically the rate-limiting step in the biosynthesis of phosphatidylcholine (PC), the most abundant lipid in eukaryotic membranes (21). A maize line containing a Mu insertion 35 bp upstream of the start codon of the *ZmCCT2* gene was defective in the later phase of gravitropism. Heterozygous and homozygous individuals displayed the defect (Fig. 2*C*). This result could be explained if the Mu insertion significantly reduced the expression of *ZmCCT2* in the heterozygous state. We measured *ZmCCT2* mRNA in the apical 5 mm of the primary root, where the gene is known to be well expressed (22, 23) by qPCR. *ZmCCT2* mRNA was significantly lower than wild type in heterozygous and homozygous Mu-insertion lines (Fig. 2*D*), thus explaining the impaired gravitropism. The QTL Chr1-2, detected in UW2, was prominent at the end of the response (Fig. 1*H*), consistent with the timing of the maize mutant phenotypes. We also measured gravitropism in a previously characterized *Arabidopsis* mutant and its Wassilewskija (Ws) wild-type background (36). The *cct2-1* mutant displayed a very minor although statistically significant difference in gravitropism (Fig. 2*E*), but not in root elongation rate (Fig. 2*F*). These results support the conclusion that natural variation in *CCT2* is responsible for gravitropism QTL Chr1-2 in maize and 4@40.3 in *Arabidopsis*.

### *ATG5* Positively Affects Gravitropism.

**Fig. 3.**
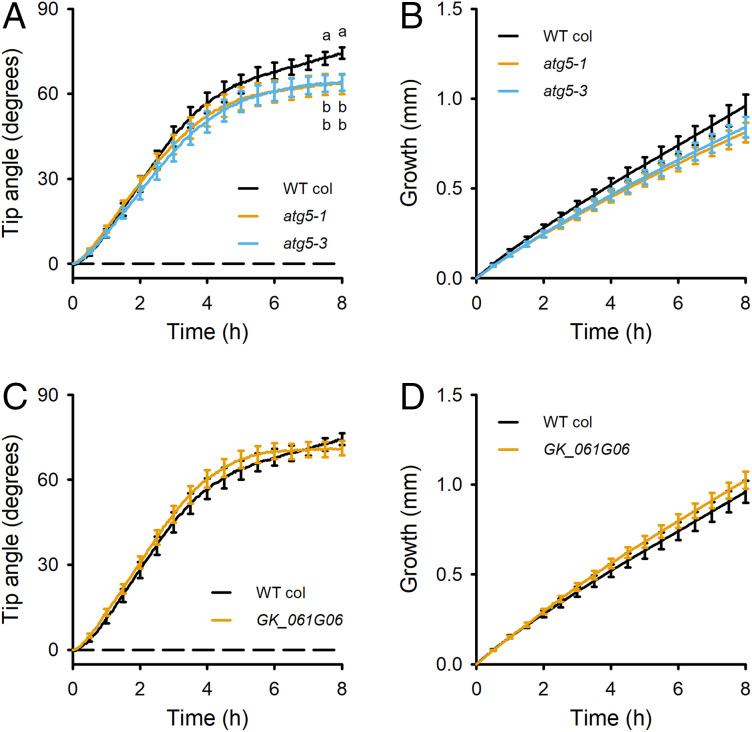
Gravitropism phenotypes support causal QTL gene status for *ATG5.* (*A*) Two independent *Arabidopsis atg5* mutants displayed impaired gravitropism in a root tip angle assay. The curves show the average of 44 wild type Col, 47 *atg5-1*, and 43 *atg5-3* seedlings. The statistically significant difference according to a one-way ANOVA with Tukey HSD test (α = 0.1) are indicated by different letters. (*B*) Elongation rate appeared to be slower toward the end of the recording period in *atg5* mutants, but the difference was not statistically significant. (*C*) A T-DNA insertion (GK_061G06 line) in the promoter region of *ATG5* did not impair gravitropism or (*D*) slow elongation. The GK_061G06 allele overexpresses *ATG5* mRNA. The curves show the average of 44 WT Col, and 47 GK_061G06 seedlings. The SEM error bars are shown at 30-min intervals rather than at each of the 240 time points for clarity.

*ATG5* encodes an autophagy-related protein best known for its role in the formation of membrane-delimited structures that bring molecules and subcellular structures to the vacuole for recycling (31). The results in Fig. 3*A* show that two different *atg5* mutants, both previously characterized as loss-of-function alleles (24, 25), displayed impaired gravitropism, but root growth during the response was not significantly affected (Fig. 3*B*). A third T-DNA insertion allele (GK_061G06) was studied. This mutant developed a greater tip angle than the wild type, in contrast to the two knockout alleles. The T-DNA in the GK_061G06 allele is very near the transcription start site of the *ATG5* gene. The T-DNA in the GK collection of mutants contains a 35S promoter for use in activation-tagging screens. We tested the possibility that the T-DNA in this case causes overexpression of the *ATG5* gene. qPCR showed that *ATG5* mRNA was 92 ± 21-fold higher (*n* = 4 biological replicates) in GK_061G06 than in wild type. If the overexpressed mRNA produces more active ATG5 protein, then this mutant further supports our conclusion that *ATG5* affects root curvature during gravitropism and that the *ATG5* gene is responsible for the Chr5-2 QTL in maize and 5@61 in *Arabidopsis*. The elongation rates were not significantly different (Fig. 3*D*).

### *ATG5* Is Linked to *UGP2*, Another Candidate Gene.

**Fig. 4.**
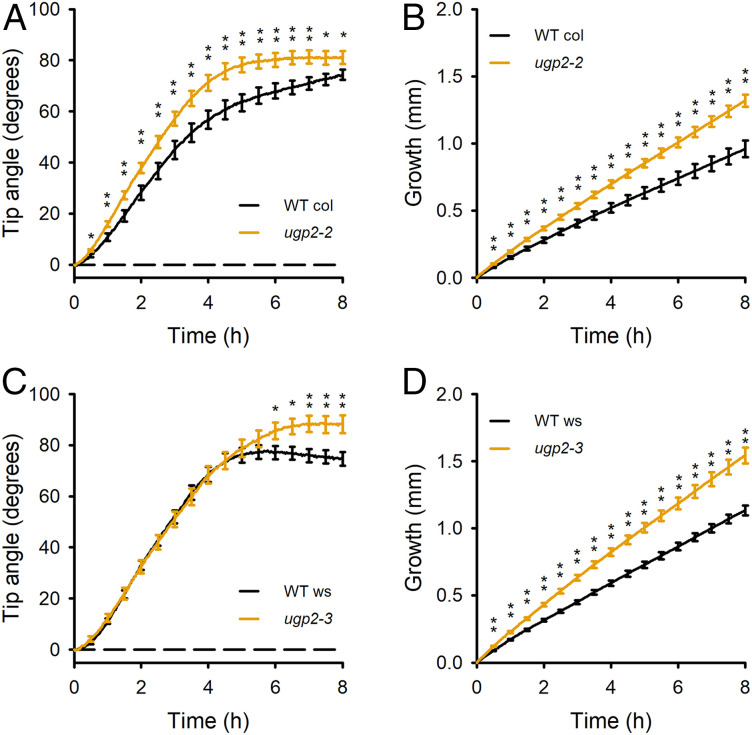
Gravitropism phenotypes support causal QTL gene status for *UGP2*. (*A*) A *ugp2* mutant in the Col-0 genetic background displayed greater gravitropism than the wild type across the time course. (*B*) The *ugp2-2* mutant also displayed faster root elongation. (*C*) A *ugp2* allele in the Ws genetic background (*ugp2-3*) displayed greater gravitropism at the end of the time course. (*D*) Elongation of *ugp2-3* roots was faster than wild type. The position of the T-DNA in the *ugp2-3* allele is not well defined, but its phenotype supports the conclusion that *UGP2* contributes to root gravitropism. The curves show the average of 44 WT (Col), 49 *ugp2-2*, 28 WT (Ws), and 24 *ugp2-3* seedlings. The SEM error bars are shown at 30-min intervals rather than at each of the 240 time points for clarity. Asterisks indicate statistically significant difference from the wild type by Student’s *t* test (*P < 0.05; **P < 0.01).

The *FAMA* and *CCT2* results (Fig. 2) are consistent with the hypothesis that the allele state of a single gene within a QTL interval affects the trait. However, within one QTL (Chr5-2 in maize, 5@61 in *Arabidopsis*), we identified two potentially causal genes using the reciprocal sequence comparison method (Table 1). One is *ATG5*, already shown in Fig. 3 to affect gravitropism. The other is *UGP2*, which encodes an enzyme that produces the form of glucose used to extend polysaccharides such as callose and cellulose (26, 27). The most straightforward hypothesis predicts that one gene but not the other would affect gravitropism. Therefore, a *ugp2* mutant would not be expected to display a gravitropism phenotype. However, a *ugp2* mutant in the Col-0 ecotype displayed enhanced gravitropism and a significantly greater rate of root elongation (Fig. 4 *A* and *B*). Another allele, *ugp2-3* in the Ws ecotype had a similar phenotype, but, unlike *ugp2-2*, PCR assays did not demonstrate the presence of T-DNA, so we could not confirm the exact nature of the allele. Nevertheless, its phenotype is consistent with the better characterized *ugp2-2* allele. Apparently, within the same QTL, *ATG5* promotes gravitropism, while *UGP2* dampens it. Natural variation in both presumably produces QTL Chr5-2 in maize and 5@61 in *Arabidopsis*.

At5g16860 is a third potential contributor to the *Arabidopsis* gravitropism QTL 5@61 because the most similar gene in maize resides within the maize gravitropism QTL Chr10-2 (Table 1). The gene, a member of the tetratricopeptide repeat (TPR)–like superfamily, has apparently not been studied before. T-DNA insertion mutants with a high likelihood of disrupted function were not available. Lack of suitable T-DNA mutants is also why we did not test the *ZYP1* candidate gene (Table 1) for a gravitropism function.

### No Apparent Role for *PIP5K* in Gravitropism.

**Fig. 5.**
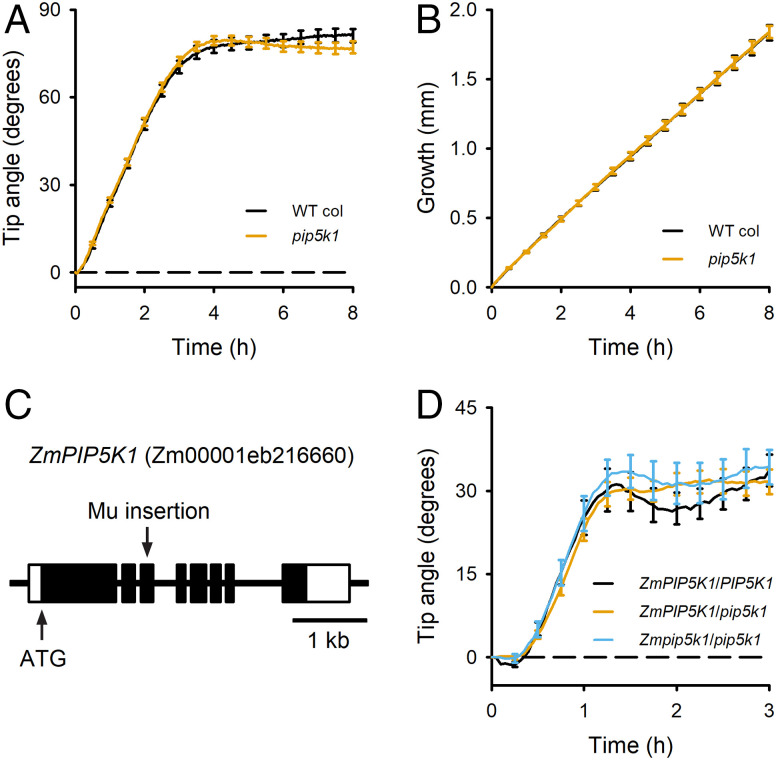
Gravitropism responses of maize and *Arabidopsis pip5k1* mutants do not support a causal QTL gene status for *PIP5K1*. (*A*) An *Arabidopsis pip5k1* mutant performed gravitropism indistinguishably from wild type. (*B*) The *pip5k1* mutation did not affect *Arabidopsis* root elongation during gravitropism. (*C*) A maize line having a Mu insertion in the third exon of the *ZmPIP5K1* gene was obtained. (*D*) Gravitropism was not significantly different from wild type in maize seedlings either heterozygous or homozygous for the mutant allele. Error bars represent SEMs. The curves show the average of 45 WT Col, 38 *pip5k1*, 26 *PIP5K1*/*PIP5K1*, 43 *PIP5K1*/*pip5k1*, and 16 *pip5k1*/*pip5k1* seedlings.

*PIP5K1*, the *Arabidopsis* At1g21980 gene, encodes a phosphatidylinositol-4-phosphate 5-kinase (28). *PIP5K1* resides in the interval of QTL 1@64. The most similar gene in the maize genome resides in the maize Chr5-1 QTL interval (Table 1). A T-DNA mutant in the Col-0 ecotype of *Arabidopsis* performed gravitropism and elongated indistinguishably from the wild type, indicating that *PIP5K1* is not the causal gene (Fig. 5 *A* and *B*). A maize line containing a Mu insertion in the third exon of *ZmPIP5K1* (Zm00001eb216660) also did not display a gravitropism phenotype, compared to the W22 wild type (Fig. 5 *C* and *D*). *PIP5K1* is a one-to-one ortholog residing in maize and *Arabidopsis* gravitropism QTL intervals that apparently does not contribute to the mapped trait.

### Four Genes Not Previously Known to Affect Gravitropism.

We conclude that *CCT2*, *UGP2*, *ATG5*, and *FAMA* are the genes responsible for the natural variation in root gravitropism that produced QTL Chr1-2, Chr5-2, and Chr8-2 in maize and 4@40.3, 5@61, and 3@17 in *Arabidopsis*, because one-to-one orthologs of these genes reside in both maize and *Arabidopsis* gravitropism QTL intervals (Table 1), and because mutations in these genes affect gravitropism (Figs. 2–4).

## Discussion

A QTL orthology approach based on two species that diverged ∼150 million years ago is more likely to identify conserved components of a mechanism than components (genes) that evolution subsequently involved in one lineage but not another. A QTL orthology approach would probably not be effective if the species being compared were more closely related and their genomes more syntenic, nor would it be expected to identify genes that participate in a process that was poorly conserved between them. Gravitropism may be especially amenable to study by a QTL orthology approach because it would have been key to the original specialization of shoots and roots after the successful colonization of land, and at least within seed plants, it appears to be based on a homologous architecture of statolith-based sensing in the root tip and auxin redistribution in a more basal growth zone (16). The QTL orthology approach may not be as successful for traits that evolved later to adapt to less constant and pervasive environmental factors.

Reciprocal Basic Local Alignment Search Tool (BLAST) for orthology identification discriminates well against genes with one-to-many or many-to-many orthology relationships, although it is prone to false-negative errors (29). An approach using more permissive, many-to-many orthology methods may identify additional gravitropism genes, although they may be more difficult to validate using single gene knockouts. The practical benefit of the reciprocal BLAST approach to identifying orthologs in the present case was that it severely reduced the candidate gene list from thousands to seven one-to-one pairs. Of these seven, we validated roles in gravitropism for *ATG5*, *CCT2*, *FAMA*, and *UGP2* (Table 1).

ATG5 plays a rate-limiting role in the formation of autophagosomes, which are membrane-delimited structures that degrade and recycle cellular components (30). A complex consisting of ATG5, ATG12, and ATG16 conjugates the phosphatidylethanolamine lipid to ATG8, which promotes autophagosome expansion and ultimate closure (31). At least two reports demonstrate a role for ATG5 in root hydrotropism, bending growth in response to external water potential gradients rather than by gravity (32, 33). Both studies show severely impaired hydrotropism in *atg5* mutants. Jiménez-Nopala et al. (33) reported that gravitropism was normal in *atg5*, but their measurement method would not have detected the subtle phenotype shown in Fig. 3*A*. The results in Fig. 3 support the present QTL-based evidence that ATG5-dependent autophagy plays a role in root gravitropism. The QTL may result from variation in the amino acid sequences of the ATG5 protein because there are three amino acid differences between the Cvi and Ler *Arabidopsis* parents and two between the B73 and Mo17 maize versions (*SI Appendix*, Table S3). ATG5 is expressed in the columella, meristem, and elongation zone cells of *Arabidopsis* roots (34) (*SI Appendix*, Table S3). The primary root of 3-d-old maize seedlings, the same stage used in this study, contains significant amounts of ATG5 mRNA (22) (*SI Appendix*, Table S3). The ATG5 protein was not detected in the maize seedling root, but it was evident in the germinating kernel (23) (*SI Appendix*, Table S3).

The CCT2 enzyme associates with the face of lipid bilayers in some membranes. Its activity maintains the characteristically high percentage of PC relative to other phospholipid species in membranes. *SI Appendix*, Fig. S1 and Table S3 show that CCT2 is expressed strongly in the meristem and root apex, where cells must synthesize membrane materials before entering a period of extraordinary expansion, reaching rates >50%/h (35). Perhaps this membrane synthesis activity plays a role in coordinating the unequal rates of cell expansion across the growth zone. Briefly chilling adult plants increases the expression of *CCT2* and PC levels in rosette leaves (36), so it may be regulated by other environmental factors, including gravity stimuli. PC has also been shown to play a long-distance signaling role by binding to the florigen protein FT to influence flowering time (37). Knocking down a *CCT* gene in *Drosophila* photoreceptor cells strongly impairs the light-triggered changes in membrane potential required for vision (38). The QTL may be due to sequence variation in the promoter because the coding regions of the Cvi and Ler alleles were the same, as were the B73 and Mo17 alleles in maize (*SI Appendix*, Table S3). Perhaps variation in *CCT2* expression in roots varies a membrane property or changes the production of a signaling factor important to gravitropism.

The FAMA bHLH transcription factor can modify root gravitropism (Fig. 2A). FAMA is mostly studied in *Arabidopsis* cotyledons, where it ceases division of stomatal cell precursors and promotes guard cell differentiation (20). A function in roots has not been previously reported. *FAMA* is not expressed in roots or only at very low levels (*SI Appendix*, Table S3). Perhaps some effect of FAMA action in cotyledons influences how roots respond to a gravity stimulus. Alternatively, a low level of FAMA in roots may directly participate in the gravitropism mechanism. This possibility is supported by the finding that FLP and MYB88, two other bHLH transcriptions that function in stomatal development at the same stage as FAMA, affect gravitropism and the expression of PIN auxin efflux transporters (39).

The QTL that contains *ATG5* also contains *UGP2*, in both maize and *Arabidopsis*. The UGP2 enzyme produces uridine diphosphate-glucose, a substrate for numerous reactions such as synthesizing sucrose, extending polysaccharides such as cellulose and callose, and glycosylating proteins. The biochemical function of UGP2 is better understood than its role in physiology or development. Neither the general composition of cell walls extracted from rosette leaves nor bulk sucrose levels in a *ugp1 μgp2* double mutant are different from wild type (26, 27). *UGP2* is highly expressed in the *Arabidopsis* and maize primary root (*SI Appendix*, Table S3), consistent with our measurements of greater root elongation (Fig. 4) and a previous report of longer roots in *ugp1 μgp2* double mutants (27). Faster elongation could cause the observed greater gravitropism (Fig. 4), but the relationship between elongation rate and gravitropism is condition dependent and nonlinear (11), so whether the *ugp2* gravitropism phenotype is a result of a higher elongation rate remains an open question. The origin of the QTL could be due to differences in the protein between the parents. In *Arabidopsis*, there are three amino acid differences between Cvi and Ler, and in maize there are two amino acid differences between B73 and Mo17. In both species, this gene has multiple gene models due to alternate splicing, but at least in Col-0, only one of the versions expresses well in the root apex (*SI Appendix*, Table S3).

Mutant analysis failed to confirm a gravitropism phenotype for PIP5K1 in either *Arabidopsis* or maize, even though the very closely related *PIP5K2* (At1g77740) has been shown to play a role in gravitropism (40), and *pip5k1pip5k2* double mutants have an even clearer gravitropism phenotype than the *pip5k2* single mutant (41). If natural variation in *PIP5K1* in maize and *Arabidopsis* is the cause of QTL Chr5-1 (1@64), then the lack of a gravitropism phenotype in *pip5k1* mutants may be due to the genetic backgrounds of the alleles (Col-0 in *Arabidopsis* and W22 in maize) being different from the populations used to map the QTL. Ideally, reverse genetic tests of candidate genes would be performed in the genetic backgrounds of the parent of the RILs used to map the QTL.

Precisely measuring gravitropism in mapping populations of two distantly related species, identifying one-to-one orthologs within the QTL intervals by reciprocal BLAST, and testing their functions by reverse genetics identified genes not previously known to affect this fundamental process. As data-sharing infrastructure and open-data practices make reusing phenotype data more feasible, this approach may be found to be generally useful, enabling two QTL studies to be much more powerful than the sum of their results.

## Materials and Methods

### Germplasm.

The maize *cct2 Mutator* transposon mutant in the B73 inbred background (mu-illumina_231033.6) was obtained from the Mu-illumina team (42) through their website (teosinte.uoregon.edu/mu-illumina/). The *pip5k1* (mu1045297) *Mu* transposon maize mutant in the W22 inbred background was obtained through the maize gene database (43). Both mutant lines were backcrossed twice to their original inbred lines, B73 and W22, respectively, and self-crossed. Segregating populations were used for phenotype analysis. Seedlings were subsequently genotyped.

### Plant Materials and Growth Conditions.

Maize kernels were submerged in continuously aerated deionized water maintained at 28 °C. All of the subsequent steps were performed in a photobiology darkroom. Imbibed kernels were mounted on a plastic strip that suspended the kernel tip-down, overlaid with wet paper (Kimwipe), and placed in a clear plastic box (16 × 16 × 3 cm). Dim red light (5 µmol m^−2^ s^−1^) was applied so that germination and photomorphogenesis would proceed in the absence of any phototropic stimuli. Recording chambers, one per camera, were prepared using square Petri dishes (13.5 × 13 × 1.5 cm) and 1% agar with no added nutrients. Removal of an upper portion of the agar in the dish produced an empty headspace and a horizontal agar ledge. Seedlings having a root between 5 and 15 mm were removed from the germination box and placed on the agar ledge of the recording plate such that the roots could grow down along the surface of the vertical agar slab. The original orientation of the primary root, which was not always vertical, was maintained as best as possible during transfer to the measuring plate. Up to eight seedlings were spaced along the ledge of one recording plate.

### Image Acquisition and Analysis.

A bank of seven Marlin F-146B charge-coupled device cameras (AVT Corporation) collected images of seedlings on seven agar plates, each held vertically in a sample holding fixture. Each camera was equipped with an 18-mm focal length lens (M0814-MP; Computar), which produced an image of the entire plate at a resolution of 10 pixels per millimeter. A longpass filter made with infrared-transmitting Plexiglas (ACRY11460; Ridout Plastics) was placed in front of the lens. A custom-built array of infrared light-emitting diodes (LEDs) having a peak output at 940 nm backlit each translucent agar plate. Each camera thereby collected an image of the seedlings using infrared radiation delivered from behind the sample. The top edge of the square-frame fixture holding the sample plates was fitted with LEDs that provided continuous dim red light (5 µmol m^−2^ s^−1^, peak output at 660 nm) to the shoot side of each kernel. The seedlings were maintained vertically in this fixture for 2 h before the plate was rotated by 90° within the frame of the fixture to induce gravitropism. The top-edge red LEDs were switched off and a row of red LEDs on the side of the frame was switched on so that red light continued to illuminate the shoot side of each seedling. A computer collected and stored images from each of the 7 cameras every 3 min for 3 h. A completely automated method identified each root tip at each time point in the series and measured its angle. The image processing and machine learning-based method is presented in *SI Appendix*, *SI Methods*.

### QTL Analysis.

Stepwise QTL analysis (17) was performed as described in Moore et al. (14). The significance thresholds were determined from the results of 25,000 permutations performed on distributed high-throughput computing resources. The qtl library (44) was used, with 256 rounds of imputation.

### Curve Fitting.

The grofit R software package (45) was used to fit the Logistic and Gompertz functions to the tip angle curves data. The grofit R software was also used for the spline fitting.

### Orthology Analysis.

BBHs (reciprocal best BLAST hits) were identified using Perl scripts to perform reciprocal protein-protein BLAST (blastp from the blastall implementation) (46) of the predicted proteins from all maize intervals against all *Arabidopsis* genome predicted proteins, and reciprocally of predicted proteins from the *Arabidopsis* QTL intervals against all maize predicted proteins. The percent identities of the proteins predicted to be encoded by the seven orthologous pairs (Table 1) were calculated using the default parameters of the blastp tool at https://blast.ncbi.nlm.nih.gov/Blast.cgi.

### Transgenic Line.

To create the pCCT2:CCT2-eGFP construct, a genomic fragment containing 1.4 kb of promoter sequence upstream of the initiation codon and the entire coding region except stop codon was amplified with restriction sites PacI and AgeI using the 5′ to 3′ oligonucleotides TTAATTAATCTGGAGATCTTCCGAGCTT and ACCGGTTTCTTTACTGTTTCAATCCCTTTG. The amplified fragment was cloned into pGEM-T-Easy vector (Promega) and its sequence was confirmed to be identical to that in the wild type, N60000 (Col-0). The verified fragment was inserted into the PacI/AgeI site of the pEGAD vector (47). *Arabidopsis* (Col-0) was transformed with the construct by floral dipping (48).

### Genotyping.

Genotyping of mutant alleles was performed by PCR with the oligonucleotide primers shown in *SI Appendix*, Table S4. For genotyping of the maize mutant alleles, genomic DNA was extracted using the cetyltrimethylammonium bromide (CTAB) method as previously described (49). For the detection of Mu insertion in maize mutants, TIR6 primer was used as a Mu insertion specific primer (50).

### qPCR Analysis.

For maize samples, the apical 5 mm of each root grown in the same condition as the gravitropism experiment was harvested individually and frozen in liquid nitrogen. Coleoptile tissue from each seedling was used to genotype each harvested root tip. Between 13 and 19 roots with the same genotype were pooled and RNA was extracted using the RNeasy Plant Mini Kit (Qiagen) with RNase-Free DNase Set (Qiagen). For *Arabidopsis*, 10–12 seedlings grown for 3 d in the same way as those used for gravitropism experiments were harvested and frozen in liquid nitrogen. The total RNA was extracted as stated above. qPCR with reverse transcription was performed with the Mx3000P qPCR system (Agilent) using the KAPA SYBR FAST one-step qRT-PCR kit (Roche). Beta-TUB (51) and ACT8 (52) were used as internal controls for maize and *Arabidopsis*, respectively. The primer sequences used are listed in *SI Appendix*, Table S4.

### Root Microscopy.

For confocal imaging, roots were stained with propidium iodide (10 µg/mL) for 5 min. pCCT2:CCT2-eGFP (enhanced green fluorescent protein) was observed by the Zeiss 780 confocal laser scanning microscope. We used the optics C-Apochromat 40x/1.1 W Korr M27 lens. The excitation-emission settings used for eGFP and propidium iodide were excitation 488 nm, emission 536 nm and excitation 561 nm, and emission 648 nm, respectively. A Zeiss AxioZoom was used to collect an image of fluorescence from the entire root. The pCCT2:CCT2-eGFP was visualized using a short-wavelength emission filter set (450–490 nm excitation, 500–550 nm emission).

### Bioinformatic Analyses.

We obtained the Ler-0 and Cvi-0 genome assemblies with their corresponding gene annotations from Jiao and Schneeberger (53). The loci containing genes At3g24140, At4g15130, At5g17290, and At5g17310 plus 1 kb downstream and upstream were retrieved with ‘bedtools getfasta’ version 2.27.1 (54) and ‘blastn’ version 2.2.29 (46) commands. The annotations of the reference genome Col-0 TAIR10 were downloaded from https://www.arabidopsis.org (file “Araport11_GFF3_genes_transposons.May2022.gff.gz”). Finally, the multiple alignment between the DNA sequences of the three accessions was carried out with MAFFT version 7 (55) and visualized with benchling (https://benchling.com).

## Supplementary Material

Supplementary File

Supplementary File

## Data Availability

All of the study data are included in the article and/or supporting information.

## Change History

September 29, 2022: The article has been updated to add an affiliation for author Il-Youp Kwak.
