# Supplementary material for: Leveraging orthology within maize and Arabidopsis QTL to identify genes affecting natural variation in gravitropism
Source: Proc Natl Acad Sci U S A. 2022 Sep 26;119(40):e2212199119. doi: 10.1073/pnas.2212199119 (PMC9546580; doi:10.1073/pnas.2212199119)
Supplement: Supplementary File [file pnas.2212199119.sapp.pdf]

PNAS

Supplementary Information for

Leveraging orthologous relationships within maize and Arabidopsis quantitative trait loci to identify new gravitropism genes

Takeshi Yoshihara, Nathan D. Miller, Fernando A. Rabanal, Il-Youp Kwak, Karl W. Broman, Hannah Myles, Boris Sadkhin, Ivan Baxter, Brian P. Dilkes, Matthew E. Hudson, Edgar P. Spalding

Edgar P. Spalding
Email: spalding@wisc.edu

This PDF file includes:

Supplementary Methods text.
Figure S1
Tables S1 to S4
Legend for Movie S1

Other supplementary materials for this manuscript include the following:

Movie S1

Supplementary Methods Text

Image processing and Hidden Markov Modeling of the seedling to identify the root tip

The first step in the method identified each seedling within each image by combining grayscale-morphology and edge-detection methods, then cropped each seedling through its times series with a box of constant dimensions. Within each cropped sub-image, a series of 100 hundred level-set curves spanning 75% of the black-to-white (0-255) range was generated. The longest closed curve in each box was taken as the seedling's contour. Each contour was converted to a standardized map by subtracting the seedling's center of mass position from each contour point.

We constructed a seven-state Hidden Markov Model (HMM) that could reliably detect the region of the contour that included the root tip, even as the tip changed orientation during gravitropism. We considered the contour to consist of seven regions in clockwise order: upper kernel, kernel-root transition, upper root, root tip, lower root, root-kernel transition, and lower kernel (Figure). Grayscale values within a circular patch (radius = 30 pixels) centered at each contour point and the patch's position and orientation relative to the kernel's center of mass were reduced by principal components analysis to produce the observations the HMM used to region-label each contour point. The model's transition functions allowed only self-connections or next-state (clockwise) progression along the seven-label sequence.

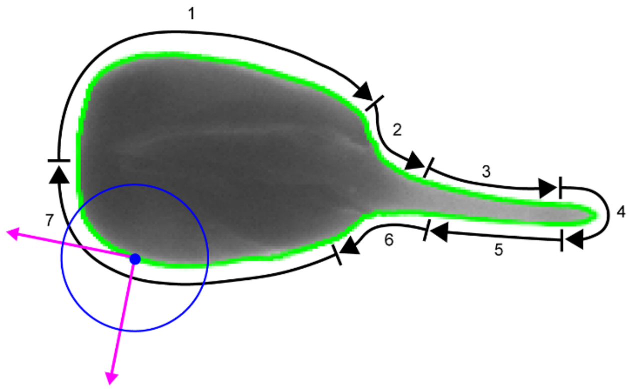

Figure. Elements of a maize seedling model constructed to locate the root tip. The seedling contour was divided into seven regions. A Hidden Markov Model was trained to identify region 4 (root tip) using grayscale information of pixels in circular patches located at each contour point, their position relative to the center of mass, and orientation information provided by vectors normal and tangent to the contour point at the center point of each patch.

To train the HMM, we used a hand-labelled set of greater than 100 seedlings. Training was initiated using labels determined by k-means clustering (k=7) to produce initial distribution parameters and transition parameters. The Viterbi algorithm for determining the most likely state assignment was iteratively applied, and the state distributions and transitions were updated until no change in the labels at each contour point was detected. Post training, the Viterbi algorithm and the trained HMM were used to label each point of each seedling's contour as one of the seven states (regions). Contour coordinates within 30 pixels of the identified tip (state 4) defined a region of the image corresponding to the root apex, which was binarized and the pixels subjected to principal components analysis. The angle of the resulting first eigen vector was recorded as the tip angle.

Supporting Figure S1

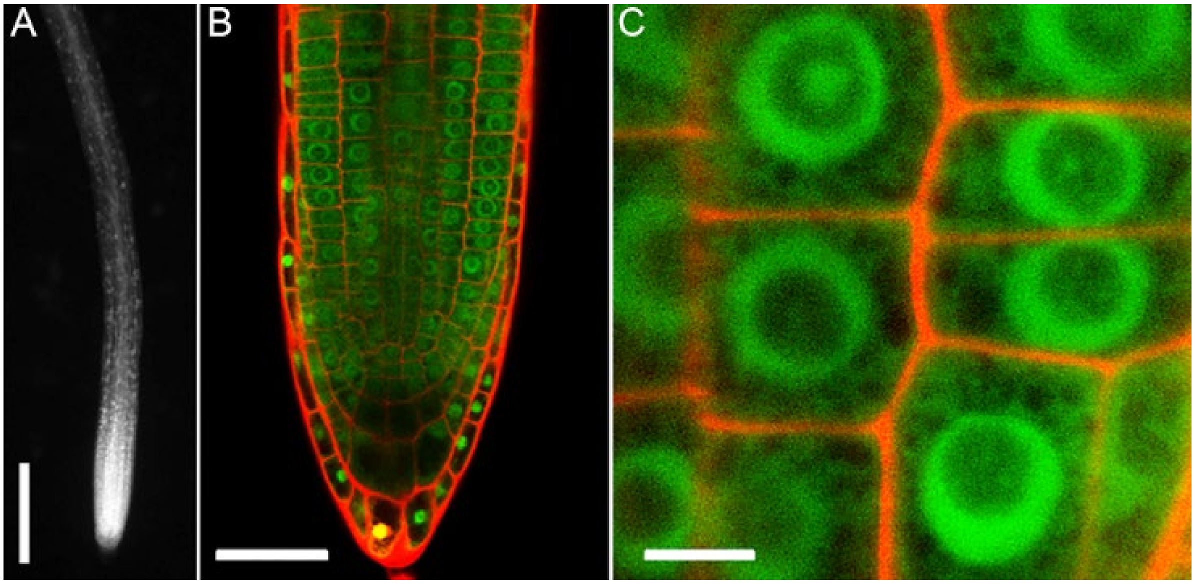

Figure S1. Arabidopsis CCT2 expression pattern and subcellular localization. A transgenic plant expressing a *pCCT2:CCT2-eGFP* reporter transgene was studied with A) epifluorescence microscope to visualize expression pattern in the root apex (bright area), or a B) a laser scanning confocal microscope for more detail in a root stained with propidium iodide to show cell boundaries in red, and C) at higher magnification to show the protein surrounding or within the nucleus and other endomembranes, which is typical of the two CCT2 proteins all eukaryotes apparently possess (Cornell and Ridgway, 2015). Scale bar is 0.25 mm in A, 40 µm in B, and 5 µm in C.

Cornell RB, Ridgway ND (2015) CTP:phosphocholine cytidylyltransferase: Function, regulation, and structure of an amphitropic enzyme required for membrane biogenesis. Prog Lipid Res **59:** 147-171

Supporting Table S1

QTL	Exp	Marker	Position (cM)	Max LOD score	Left interval border	Right interval border	Number of genes in interval
Chr1-1	UW1	umc1397	226.4	6.0	umc1403	AY110028	260
	UW2	AY110052	229.6	7.4	bnlg1484	AY110028	
Chr1-2	UW2	mmp101	441.2	3.7	ufg43	umc1906	1018
Chr1-3	UW2	cdo87b(ptk)	1006.9	6.6	AY110426	hon110	214
Chr2-1	UW2	mmp116	482.2	3.8	mmp177b	bcd808c	532
Chr3-1	UW1	umc2261	210.0	6.8	mmp36	mmp29	1187
	UW2	umc1742	189.0	4.9	nfc104c	umc1527	
	FL	jpsb527a	283.9	7.6	umc2264	umc1693	
Chr5-1	UW2	tua4	147.5	4.2	rz630f(sat)	csu554b(rnh)	165
Chr5-2	FL	bnlg1902	297.5	9.3	isu61e	umc2298	326
Chr5-3	UW2	umc2303	408.8	6.9	umc1264	csu173	142
Chr7-1	UW1	AY104465	69.1	5.5	asg8(myb)	AW308691	76
Chr7-2	UW2	c7.loc470	470.0	6.0	bcd349	umc1768	125
	FL	umc1768	481.1	7.2	csu8	bnlg2259	
Chr8-1	UW2	bcd1823a	135.6	7.3	mmp57	npi585a	145
Chr8-2	UW2	cdo202e(mcf)	224.8	9.6	umc1910	AY110032	311
Chr9-1	UW2	c9.loc30	30.0	9.3	bnlg2122	ufg41	86
Chr9-2	UW1	umc1789	534.2	8.3	nfd104d	phi448880	140
	UW2	umc1789	534.2	6.6	isu49	AY109543	
Chr10-1	UW2	umc1453	274.4	5.7	umc64a	php15013	625
Chr10-2	UW2	ufg15	444.8	8.6	ufg15	mmp181	120

Table S1. Time dependent QTL (tQTL) for gravitropic response in maize roots.

Exp, the experiment (population) in which the tQTL were found; Marker, the marker nearest the LOD peak; Position, the position of the LOD peak; Left and right interval border, the markers nearest to the left and right borders of the QTL at a position 1.5 LOD score below the peak; Number of genes in interval, number of genes found between the left and right borders of the 1.5 LOD interval.

Supporting Table S2

Exp	Fitting model	Parameter	Chromosome	Marker	Position (cM)	LOD	1.5 LOD interval		Overlapped QTL
							Left	Right	
UW1	Logistics	mu	1	c1.loc313	227.7	2.9	mmp66	c1.loc313	Chr1-1
			9	mmp168	486.5	4.3	c9.loc480	ay109543	Chr9-2
		lambda	null						
		A	1	c1.loc198	195.7	3.6	c1.loc187	c1.loc219	Chr1-1
			7	c7.loc70	70.0	4.5	c7.loc62	c7.loc79	Chr7-1
		Integral	7	ay104465	69.1	4.4	c7.loc63	c7.loc80	Chr7-1
	Gompertz	mu	1	c1.loc229	226.7	5.2	mmp66	c1.loc290	Chr1-1
			9	c9.loc535	535.0	4.7	c9.loc481	ay109543	Chr9-2
		lambda	3	umc2261	210.4	3.7	c3.loc202	c3.loc288	Chr3-1
		A	null						
		Integral	1	c1.loc229	226.7	4.0	c1.loc215	c1.loc312	Chr1-1
			7	c7.loc69	69.0	4.5	c7.loc63	c7.loc81	Chr7-1
	Spline	mu	1	c1.loc289	287.6	4.3	np1439a	c1.loc319	Chr1-1
		lambda	3	umc2261	210.4	4.1	c3.loc201	chr126b	Chr3-1
			9	c9.loc521	521.0	3.9	c9.loc498	phi448880	Chr9-2
		A	7	c7.loc70	70.0	4.1	c7.loc62	c7.loc81	Chr7-1
		Integral	1	c1.loc229	226.7	4.0	c1.loc215	c1.loc312	Chr1-1
			7	c7.loc69	69.0	4.5	c7.loc63	c7.loc81	Chr7-1
		Spline minus Logistics	9	umc1789	534.2	3.1	c9.loc312	ay109543	Chr9-2
		Spline minus Gompertz	7	c7.loc49	49.0	5.2	c7.loc35	c7.loc52	
			9	c9.loc522	522.0	1.8	c9.loc1	aw216329	Chr9-2
		Growth rate	null						
UW2	Logistics	mu	1	c1.loc235	232.7	4.5	ay109929	c1.loc330	Chr1-1
			3	umc2117	190.2	3.8	c3.loc182	ay110352	Chr3-1
			8	c8.loc135	135.0	4.9	c8.loc115	chr117a	Chr8-1
			10	c10.loc275	275.0	5.3	c10.loc243	ay109698	Chr10-1
		lambda	8	c8.loc274	274.0	6.0	bngl2082	umc1457	Chr8-2
		A	8	c8.loc224	224.0	7.4	c8.loc218	c8.loc250	Chr8-2
		Integral	1	bngl1866	290.1	4.3	umc1397	c1.loc453	Chr1-1
			5	c5.loc144	144.0	4.1	c5.loc132	csu554b(rnh)	Chr5-1
			8	c8.loc224	224.0	7.0	c8.loc219	umc1984	Chr8-2
			10	umc1453	274.4	4.2	c10.loc250	umc1084	Chr10-1
	Gompertz	mu	1	c1.loc234	231.7	5.7	umc1397	c1.loc243	Chr1-1
			5	c5.loc160	160.0	4.5	c5.loc141	c5.loc164	Chr5-1
			8	cdo328	159.2	4.2	c8.loc129	c8.loc171	Chr8-1
		lambda	8	c8.loc276	276.0	6.3	umc32b	c8.loc309	Chr8-2
			9	umc1789	534.2	3.7	c9.loc292	aw216329	Chr9-2
		A	8	c8.loc224	224.0	7.9	c8.loc218	c8.loc250	Chr8-2
		Integral	5	c5.loc143	143.0	4.1	c5.loc130	c5.loc178	Chr5-1
			8	umc2355	232.9	6.1	c8.loc219	c8.loc251	Chr8-2
	Spline	mu	3	c3.loc189	189.0	5.2	nfc104c	umc1655	Chr3-1
			8	bcd1823a	135.6	6.5	c8.loc113	mmp166	Chr8-1
			10	umc1453	274.4	5.0	c10.loc243	umc1084	Chr10-1
		lambda	8	c8.loc286	286.0	5.9	c8.loc230	c8.loc309	Chr8-2
			9	c9.loc305	305.0	4.0	c9.loc286	c9.loc318	
			9	umc1789	534.2	6.8	c9.loc509	ay109543	Chr9-2
		A	8	bcd1823a	135.6	3.8	c8.loc129	mmp166	Chr8-1
			8	c8.loc223	223.0	6.0	c8.loc218	c8.loc250	Chr8-2
			10	umc1453	274.4	4.7	c10.loc260	c10.loc303	Chr10-1
		Integral	1	bnlg1866	290.1	4.3	c1.loc229	c1.loc453	Chr1-1
			5	c5.loc144	144.0	4.2	c5.loc132	csu554b(rnh)	Chr5-1
			8	c8.loc224	224.0	7.1	c8.loc219	umc1984	Chr8-2
			10	umc1453	274.4	4.3	c10.loc250	umc1084	Chr10-1
		Spline minus Logistics	2	c2.loc421	421.0	4.5	c2.loc415	c2.loc427	Chr2-1
			4	umc2139	574.8	3.6	c4.loc573	c4.loc638	
			7	c7.loc180	180	7.5	c7.loc179	c7.loc181	
		Spline minus Gompertz	9	umc1789	534.2	4.0	c9.loc510	ay109543	Chr9-2
		Growth rate	1	aw400087	287.2	4.6	c1.loc285	c1.loc337	Chr1-1
			4	c4.loc397	392.9	4.2	c4.loc390	umc19	
			5	c5.loc392	392. 0	5.5	bnl571a	c5.loc400	Chr5-2

Supporting Table 2. Maize parameter QTL (pQTL) derived from fitting the indicated model to root tip angle time courses. The columns report the position, significance, and correspondence (overlap) with the maize tQTL in Supporting Table 1.

Supporting Table S3.

	ARABIDOPSIS		MAIZE	
	Cvi vs Ler differences	Expression in Col-0 primary root*	B73 vs Mo17 differences	Expression in B73 primary root 3 DAG**
FAMA<br><br>At3g24140<br>GRMZM2G162450	8 SNPs and 2 indels in UTRs, including an 8 bp indel immediately before the start codon; 3 SNPs and an indel in introns; 2 synonymous SNPs in exons; no differences in predicted amino acid sequences	columella 0<br>meristem 0.05<br>elongation 0.01	No SNPs in genomic DNA of gene model	Not detected
CCT2<br><br>At4g15130<br>GRMZM2G132898	7 SNPS in UTRs; no SNPs in introns; no differences in predicted amino acid sequences	columella 16.9<br>meristem 17.4<br>elongation 19.9	No SNPs in genomic DNA of gene model	272,<br>protein is highest in seedling root meristem and elongation zone
ATG5<br><br>At5g17290<br>GRMZM2G098420	8 SNPS and an indel in UTRs; 48 SNPs and 3 indels in introns; 4 synonymous SNPs in exons; 3 SNPs that change amino acids T to R in CDS8, V to E in CDS7, and R to K in CDS7	columella 7.5<br>meristem 8.1<br>elongation 9.7	There are 25 SNPS and 6 indels between the B73 and Mo17 gene. The B73 gene produces 4 transcripts. If translated, they produce proteins of 46, 153, 216, or 374 amino acids. Based on transcript sequences, 4 amino acid differences are predicted between the B73 and Mo17 versions of the longest (canonical) protein.	31,<br>protein detected in germinating kernel and in endosperm, not detected in seedling root
UGP2<br><br>At5g17310<br>GRMZM2G098370	13 SNPs and 3 indels in introns. Cvi cannot produce 2 of the 4 alternate gene models in the TAIR10 reference sequence; 3 SNPs change amino acids D to N in CDS6, R to P in CDS12, and V to A in CDS14 in the At5g17310.2 model	columella 8.9<br>meristem 11.6<br>elongation 20.0<br><br>These values are for the At5g17310.2 model. Expression of the other models did not exceed 1.	There are 57 SNPs and 12 indels between the B73 and Mo17 gene. The B73 gene produces 3 different transcripts. If translated, they produce proteins of 231, 294, or 467 amino acids. Two amino acid differences are predicted between the B73 and Mo17 versions of the longest (canonical) protein.	542,<br>protein detected in root elongation zone and meristem 5 d after germination

Supporting Table S3. Summary of sequence polymorphisms between parental alleles and expression levels in a reference genotype for the four candidate genes identified in this study.

*transcript abundance data in units of FPKM from Li et al. (2016).

Li S, Yamada M, Han X, Ohler U, Benfey PN (2016) High-resolution expression map of the Arabidopsis root reveals alternative splicing and lincRNA regulation. Developmental Cell **39**: 508-522

In the case of *FAMA*, the following transcriptomics data set was examined for evidence of *FAMA* expression, but none was found. Ryu KH, Huang L, Kang HM, Schiefelbein J (2019) Single-Cell RNA sequencing resolves molecular relationships among individual plant cells. Plant Physiology **179**: 1444–1456

**transcript abundance data in units of FPKM from Stelpflug et al. (2016).
Stelpflug SC, Sekhon RS, Vaillancourt B, Hirsch CN, Buell CR, de Leon N, Kaeppler SM (2016) An expanded maize gene expression atlas based on RNA sequencing and its use to explore root development. The Plant Genome **9**: plantgenome2015.04.0025.

Protein abundance data from Walley et al. (2016).
Walley JW, Sartor RC, Shen Z, Schmitz RJ, Wu KJ, Urich MA, Nery JR, Smith LG, Schnable JC, Ecker JR, Briggs SP (2016) Integration of omic networks in a developmental atlas of maize. Science **353**: 814-8.

The data were accessed from MaizeGDB as described by Woodhouse et al. (2021).

Woodhouse MR, Cannon EK, Portwood JL, Harper LC, Gardiner JM, Schaeffer ML, Andorf CM. (2021) A pan-genomic approach to genome databases using maize as a model system. BMC Plant Biology **21**: 385.

Supporting Table S4

Use	Gene ID	Gene name	Mutant name	Primer name	Sequence (5' -> 3')
RT-qPCR	Zm00001eb025170	*ZmCCT2*		CCT2 Fw-4	CAGGCCAAGATGCTGTTCCC
	Zm00001eb025170	*ZmCCT2*		CCT2 Rv-4	TCTTGCCCTTGTAGCGGTAG
	Zm00001d013612	*b-TUB*		b-TUB Fw	CTACCTCACGGCATCTGCTATGT
	Zm00001d013612	*b-TUB*		b-TUB Rv	GTCACACACACTCGACTTCACG
	At5g17290	*ATG5*		ATG5 F-5	GACCTCTGTCATGAACGGTGATCTT
	At5g17290	*ATG5*		ATG5 R-5	GCACAACTTGTTGAGATTGTGGAGA
	At1g49240	*ACT8*		At1g49240_qPCR-F	TCAGCACTTT CCAGCAGATG
	At1g49240	*ACT8*		At1g49240_qPCR-R	CTGTGGACAA TGCCTGGAC
Genotyping		*Mutator*		TIR6	AGAGAAGCCAACGCCAWCGCCTCYATTTCGTC
	Zm00001eb025170	*ZmCCT2*	S137-3	MuII_231033.6-1 F	GATCCGCGCCTTTTTATTG
	Zm00001eb025170	*ZmCCT2*	S137-3	MuII_231033.6-2 R	GGGGAAAATGAGAGATGCTTT
	Zm00001eb216660	*ZmPIP5K1*	UFMu-05807	Mu1045297-2 F	TCTGGTTTTGGGAGAGGATG
	Zm00001eb216660	*ZmPIP5K1*	UFMu-05807	Mu1045297-2 R	TCCATTAAGGGGCTTCACAC
	At1g21980	*PIP5K1*	*pip5k1* (SALK_146728c)	SALK_146728c LP-1	TTCCACCTGAAATCCACTGAC
	At1g21980	*PIP5K1*	*pip5k1* (SALK_146728c)	SALK_146728c RP-1	AAGATGGGTGCATGTACGAAG
	At3g24140	*FAMA*	*fama-1*	SALK_100073 LP-1	TCATTCATTTGCTTCCTACGG
	At3g24140	*FAMA*	*fama-1*	SALK_100073 RP-1	CAATACAAAAAGCTCCCCTCAC
	At4g15130	*CCT2*	*cct2-1*	AtCT2-13	CCATGACTGCTTTTGATTTCTGATGCATT
	At4g15130	*CCT2*	*cct2-1*	AtCT2-15	TATCAGTGTTCAAAGATGGCCGACCTATG
	At5g17290	*ATG5*	*atg5-1*	atg5-1 LP1	ACGTTAGCCACCAACAGATTAAGCAGTGT
	At5g17290	*ATG5*	*atg5-1*	atg5-1 RP1	CAATTCACAGATGGATTGTAACTGCAGAG
	At5g17290	*ATG5*	*atg5-3* (SALK_020601)	SALK_020601 LP-1	AAAGACCACAGAACCCGAAAC
	At5g17290	*ATG5*	*atg5-3* (SALK_020601)	SALK_020601 RP-1	CCAAATTGAATCTTCACCAGG
	At5g17290	*ATG5*	GK_061G06	GABI_061G06 LP-1	TTACCGTGAGATTCCATGGTC
	At5g17290	*ATG5*	GK_061G06	GABI_061G06 RP-1	TGCTTTGAAATGAAGGCAAAG
	At5g17310	*UGP2*	*ugp2-1* (SALK_119405)	SALK_119405 LP-1	GAAGCTTAATGGAGGTTTGGG
	At5g17310	*UGP2*	*ugp2-1* (SALK_119405)	SALK_119405 RP-1	TGCCACTGTTCATGAGAGATG

Table S4. DNA sequences of primers used for PCR

Movie S1 (separate file). A video showing the gravitropic response of seedlings of an arbitrarily selected RIL. The movie shows the complete 180 min recording period (61 images each 3 min apart).
